# In Vitro Activation of Seed-Transmitted Cultivation-Recalcitrant Endophytic Bacteria in Tomato and Host–Endophyte Mutualism

**DOI:** 10.3390/microorganisms7050132

**Published:** 2019-05-14

**Authors:** Sadiq Pasha Shaik, Pious Thomas

**Affiliations:** Division of Biotechnology, Endophytic and Molecular Microbiology Laboratory, ICAR-Indian Institute of Horticultural Research, Hessaraghatta Lake, Bengaluru 560089, India; sadiq.shaikk@gmail.com

**Keywords:** bacterial activation, cultivation recalcitrant endophytic bacteria (CREB), in vitro plant cultures, host–microbe interactions, plant growth promotion, plant tissue culture, seed transmission, *Solanum lycopersicum* L., viable but non-cultivable (VBNC) bacteria

## Abstract

This study was aimed at exploring seed transmission of endophytic bacteria in tomato utilizing aseptic in vitro conditions. Cultivation-based studies were undertaken on two tomato cultivars “Arka Vikas” and “Arka Abha” employing surface sterilized seeds, aseptically germinated seeds and in vitro grown seedlings at different stages. *Bacillus* sp. appeared primarily as seed externally-associated bacteria. Tissue homogenate from extensively surface-sterilized seeds, day-3 germinating seeds, or 10-day in vitro seedlings did not show any cultivable bacteria on two bacteriological media. Indexing of 4-week old healthy seedlings with seed-coat removal following seed germination showed bacterial association in 50–75% seedlings yielding 10^6^–10^7^ cfu g^−1^ tissues. Four endophytic bacteria appeared common to both cultivars (*Kosakonia, Ralstonia, Sphingomonas, Sphingobium* spp.) with three additional species in “Arka Abha”. The bacterial strains showed a manifold increase in growth with host-tissue-extract supplementation. Seed inoculations with single-isolates stimulated germination or enhanced the seedling growth coupled with the activation of additional endophytic bacteria. In vitro seedlings upon recurrent medium-indexing over eight weeks showed gradual emergence of endophytic bacteria. The study reveals the seed internal colonization by different bacterial endophytes in a cultivation-recalcitrant form, their activation to cultivable state during seedling growth and transmission to seedlings with mutualistic effects.

## 1. Introduction

Endophytes include mainly bacteria and fungi that colonize plants internally without any apparent negative effects on the host [1,2]. Endophytic bacteria have been isolated from various plant organs with the root-endosphere recognized as the primary niche displaying maximum colonization and diversity [3,4]. Awareness about bacterial endophytes gained more momentum during the present decade with the recognition of their potential in plant growth promotion, biocontrol, stress alleviation, and other areas [3,5], possibly serving as potential plant immune systems on account of their internal colonization [6,7].

Tomato (*Solanum lycopersicum* L.) is a seed-propagated, short duration, widely consumed, and highly popular vegetable crop the world over [8]. There is a great interest in the use of endophytic microorganisms for the biocontrol of pathogens and in organic cultivation of tomato to minimize environmental hazards and chemical residues [9,10]. In this respect, isolation and characterization of endophytic bacteria with plant growth promotion and/or pathogen control potential is highly desirable [8]. For the practical exploitation of plant growth-promoting bacteria and biocontrol agents, seed fortification forms the best form of delivery [11]. Seed transmission of endophytic bacteria would be greatly advantageous allowing the early establishment and colonization of germinating seeds by the desired organisms with minimal competition from soil microorganisms [4,12].

The traditional understanding is that endophytes are acquired by plants primarily from soil through roots/root hairs [2,3]. Seed transmission of endophytes was suggested long ago [1,13]. *Bacillus* spp. formed the most common seed-associated bacteria across plant species [4,12,13]. As for tomato, a study of the cultivable bacterial communities inside the seeds of four commercial varieties showed five *Bacillus* species [14]. Another study of bacteria within seeds of two different tomato cultivars employing 16S rRNA gene V1–V3 profiling versus cultivation showed the association of diverse bacteria with considerable variations in the community structure of the two genotypes [15]. While the cultivable communities were particularly rich in spore-forming bacteria (*Bacillus, Paenibacillus* spp.), the cultivation-independent approach did not show any *Bacillus* spp. where other Firmicutes were documented. Of late more evidence is emerging on seed-associated and seed-transmitted endophytic bacteria [16,17,18] with particular interest on vertically transmitted bacterial endophytes [8,19,20]. As of now, there is a gap in our understanding about seed-associated and vertically transmitted bacteria [19,21,22]. A recent study employing tomato seeds over two successive generations suggested vertical transmission of beneficial endophytes [8]. Most of the studies on seed endophytes employed surface sterilized seeds without separating the seed coat part. A study on wheat seeds segregating the endosperm and the embryo showed endophytic bacteria in the former but not in the embryos [23].

Soil grown seedlings fail to offer a clear conclusion about the seed transmission of plant-associated microorganisms due to the potential entry of environmental microorganisms. It warrants proper checks to ensure that the microorganisms are not remnants from inefficient surface sterilization or acquired from the surroundings. The tissue culture system offers the scope for studying plant–microbial associations guarding against external microorganisms [7]. Further, the embryo in tomato is firmly attached to the seed coat making it difficult to separate. Tomato seeds cultured in vitro show seed coat separation at germination. This ability to raise the seedlings axenically offers the scope for studying seed internally associated bacteria [24]. The plant tissue culture system has proven to be useful to isolate several uncommon bacteria arising from the activation of originally uncultivable bacteria [25,26]. Further, the cultivation of such endophytic bacteria assumes significance for assessing their functional roles and practical exploitation in agriculture. This study was envisaged to explore the seed transmission of endophytic bacteria in tomato using aseptically grown seedlings and to assess the possible mutualistic effects between seed endophytes and the host under controlled conditions. We hypothesized that utilizing the protected in vitro conditions it would be feasible to study the seed endophytes away from external and interfering microorganisms. Adopting cultivation-based approaches and the tissue culture system, the study brings out the seed internal colonization by different bacterial endophytes in tomato in a cultivation-recalcitrant form, their activation to cultivable state during in vitro seedling growth and transmission to the seedlings with significant influence on seedling development.

## 2. Material and Methods

### 2.1. Seed Material and Experimental Set Up

Three seed lots of tomato (*Solanum lycopersicum* L.) cultivars “Arka Vikas” and “Arka Abha” (ICAR-Indian Institute of Horticultural Research, Bengaluru) were employed in the cultivation-based studies. Seeds packaged in aluminium lined paper bags and stored at 4 °C or 15–16 °C were monitored for the initial bacterial load and the effectiveness of surface sterilization by plating the pre- and post-sterilization wash solutions on nutrient agar (NA). Detailed studies were undertaken using a seed lot that was in storage at 4 °C for ≥ 6 months unless mentioned otherwise. Seed bacterial associations were explored broadly at five levels: employing (i), surface sterilized seeds directly; (ii), aseptically germinating seeds, (iii), in vitro grown seedlings at cotyledonary stage (day-10), (iv), one-month-old in vitro seedlings, and (v), about 7–8 week old in vitro seedlings. Care was exercised to avoid all external microorganisms as described elsewhere [27].

### 2.2. Seed Surface Sterilization

Surface sterilization step for all the experiments, unless stated differently, involved vigorous shaking of about 100 seeds in 10 mL autoclaved distilled water (ADW) thrice followed by 5 min rinsing in 0.01% Tween-20 and three subsequent ADW washes. After 1 min ethanol (70%) treatment and a rinse in ADW, seeds were shaken for 6–7 min in NaOCl (4% available chlorine) and rinsed six times in filter-sterilized ADW (FDW). In all trials, 10 μL lot wash solutions after each step were spotted on nutrient agar (NA) and the last wash solution (400 μL) was tested on NA through spotting-and-tilt-spreading (SATS) [28] to assess the initial bacterial load and the effectiveness of surface sterilization treatments. NA plates were sealed in sterile polypropylene (PP) bags pre- and post-plating to avoid external organisms. Bacterial cfu was assessed after 2–4 days of plating. Disinfected seed batches showing bacterial colony growth from any of the last three wash solutions were not used in the subsequent studies unless mentioned differently.

For in vitro seed culturing/seedling raising, Murashige and Skoog (MS) medium [29] gelled with 3 g L^−1^ phytagel (Sigma Chemical Co., St. Louis, Mo, USA) was employed with no sugar (sucrose-minus) or hormonal supplementations unless mentioned differently. This was based on the preliminary observations that a share of seeds cultured in sucrose-plus MS medium tended to show active fungal and/or bacterial growth (5–20% cultures in different batches), and thus to avoid the chances of any bias towards fast multiplying organisms.

### 2.3. Testing Homogenate from Seeds and Germinating Seeds for Cultivable Bacteria

Surface sterilized seeds (100 no) of “Arka Vikas” and “Arka Abha” with bacterial monitoring as above were homogenized in a sterile mortar in 10 mL FDW and the seed homogenate (100 μL/seed) was applied through SATS on NA employing three replications at 10^0^ to 10^4^ dilutions and through Single Plate-Serial Dilution Spotting (SP-SDS) [30] of 10^0^ to 10^5^ dilutions on trypticase soy agar (TSA). The plates were observed for up to 7 days (37 °C for one night and thereafter at 30 °C) for any colony growths. The same was repeated with the two other seed lots.

Next, surface sterilized “Arka Vikas” and “Arka Abha” seeds (50 each) were cultured singly in glass tubes (150 × 25 mm) containing 12 mL MS medium. Once the seeds germinated giving 10–20 mm radicle (day-4), the sprouted seeds were homogenized and the homogenates were tested for bacterial cfu as above.

### 2.4. Monitoring the Cotyledonary Stage In Vitro Seedlings for Bacterial Association

Surface sterilized seeds of the two cultivars were cultured on MS medium (single seed per culture tube; 50 seeds/treatment) and incubated at 26–28 °C under 16/8 h light/dark cycle. Visibly clean seedling cultures after 7 days were indexed for any cultivable bacterial association by transferring traces of culture medium to NA/TSA employing a sterile 200 μL tip as described elsewhere [31]. Cultures showing bacterial growth on the bacteriological medium over 2–7 days were classified as bacteria index-positive (BIP) and others as bacteria index-negative (BIN). By day-10, when all the seedlings proved consistently BIN, 25 seedlings each of “Arka Vikas” and “Arka Abha” were harvested, homogenized aseptically in FDW (10 mL g^−1^ tissue) excluding the seed coat and the tissue homogenate (TH) was tested through SATS on NA, and as per SP-SDS on TSA employing four replications.

### 2.5. Testing One Month Old In Vitro Seedlings

“Arka Vikas” and “Arka Abha” seedlings initiated in a different batch on MS medium were employed. Visibly clean seedlings excluding a few that exhibited fungal growth were indexed for any bacterial association after 2- and 3-weeks of culturing as above. Indexing was repeated after 4-weeks and the seedlings were segregated to BIP and BIN categories.

### 2.6. Isolation and Identification of Endophytic Bacteria from BIP Seedlings

BIP seedlings of “Arka Vikas” (5 nos) and “Arka Abha” (20 nos) were gathered 30 days after the initial seed culturing excluding the empty seed coat. The root and shoot tissues were rinsed separately in FDW containing 0.01% Tween-20, followed by three rinses in FDW with the direct monitoring of 100 μL wash solutions. The root and shoot TH were tested through SATS and SP-SDS as above. The culture medium was also monitored after dispersing 1 g in 10 mL FDW along with the uninoculated MS medium of the same batch as control. The cfu of major bacterial associates were assessed and the distinct colony morphotypes, based on their dominance, were selected (four from BIP “Arka Vikas” and eight from BIP “Arka Abha” seedlings). The isolates were taken through three rounds of SP-SDS for single colony selection and were identified based on 16S rRNA gene homology analysis using the primers 27F and 1492R-Y as described elsewhere [27]. The empty seed shells were also monitored for bacterial associations through the SP-SDS of the homogenate.

### 2.7. Isolation and Identification of Endophytic Bacteria from BIN Seedlings

The BIN seedlings identified as per day-28 bacterial indexing were again subjected to medium-indexing on day-32 which endorsed their BIN status. By day-35, root and shoot tissues were processed separately as for the BIP seedlings and the TH were tested on NA/TSA as above. The empty seed shells were also monitored for the bacterial associations. Two colony morphotypes retrieved from BIN-“Arka Vikas” and two from BIN-“Arka Abha” seedling tissues were carried forward and identified as above.

### 2.8. Growth Assessment of Bacterial Isolates and Screening for Plant Growth Promotion (PGP) Characteristics

Selected bacterial isolates from BIP and BIN seedlings were tested for the ability to grow on MS medium (without or with 3% sucrose) in comparison with NA and TSA by spotting 0.1 OD_600nm_ samples from day-2 NA cultures prepared in FDW (2 μL) and assessing the growths after 2–7 days. Based on the similarity in colony characteristics and the identification results, five distinct organisms from “Arka Vikas” (“Av” isolates) and eight from “Arka Abha” (“Ab” isolates) were finally selected across BIP and BIN seedlings. These 13 isolates were assessed for Gram reaction, cell characteristics, cfu of 0.1 OD_600nm_ anchored (10^0^) stock [30] and for PGP characteristics [10].

### 2.9. Assessing the Endophyte-Host Reliance Employing Host Tissue Extract

The host reliance of the endophytes or a possible benefit derived by the endophytes from the host was assessed through host tissue extract (HTE) supplementation of MS-based growth medium. HTE was prepared from 4-week old BIN in vitro seedlings (1 g mL^−1^ FDW) after filter-sterilizing the TH with a 12,000× *g* spin to remove tissue debris. Sucrose-minus MS medium (2 mL) in 5 mL sterile PP tubes was supplied with 250 μL FDW (control) or HTE from “Arka Vikas” (10% v/v final), and inoculated with 250 μL of 0.1 OD inoculums from the “Av” isolates. A similar trial was undertaken with the HTE from “Arka Abha” against the “Ab” isolates. Bacterial growth was assessed (OD_600nm_) after 48 h at 28–30 °C (160 rpm) employing four replications per treatment. The experiment was repeated once.

### 2.10. Assessing the Endophyte-Mediated Host Benefits In Vitro Trials

Surface-sterilized seeds of “Arka Vikas” were treated with 2% Na_2_S_2_O_3_ (10 min) to remove any residual chloramines (that might affect the inoculants), rinsed thrice in FDW and inoculated using 0.1 OD bacterial inoculums from the five “Av” isolates (approx. 10^7^ to 10^8^ cfu mL^−1^) employing 10 μL per seed (50 seeds per isolate). The control set was treated with FDW. The same was undertaken with the eight “Ab” isolates on “Arka Abha” seeds. After 1 h, the excess inoculum was removed and 30 seeds were placed in 12 × 12 × 1.2 cm plates (HiMedia Laboratories, Mumbai, India) provided with 60 mL phytagel-gelled MS medium with 10 seeds per row forming one replication. The plates were sealed with cling film, incubated under 16/8 h light/dark conditions (26–28 °C), and monitored for seed germination. Seedling growth characteristics (shoot height, root and shoot weights) were recorded after 2 weeks. The final conclusion was derived based on seedling vigour index (SVI) as a function of seedling output and gross seedling weight [10]. The experiment was repeated twice.

### 2.11. Testing the Re-Colonization Efficacy by Endophytic Bacterial Isolates

Surface sterilized seeds of “Arka Vikas” and “Arka Abha” (with the monitoring of wash solutions) were each inoculated with the five different “Av” isolates using 0.1 OD culture for 1 h and cultured in MS medium in 12 × 12 × 1.2 cm plates (30 seeds per isolate). FDW soaked seeds served as control. Two weeks into culturing, the seedlings were tested for bacterial colonization directly, or after surface sterilization followed by Na_2_S_2_O_3_ detoxification. TH from the seedlings was tested through SP-SDS on NA to assess gross versus internal tissue colonization.

### 2.12. Monitoring of In Vitro Cultures at Different Phases of Growth and In Vitro Testing of Different Seed Lots

Visibly clean seedlings of “Arka Vikas” and “Arka Abha”, cultured singly in MS medium in 150 × 25 mm tubes as above (50 seeds each) were indexed for any bacterial association after 2, 3, or 4 weeks and the BIP seedlings were discontinued thereafter. After 6 weeks into in vitro culturing, BIN seedlings were indexed again. Both BIP and BIN seedlings were assessed for growth characteristics after 7 weeks of original culturing. Root tissues were assessed for bacterial association (cfu g^−1^) through SP-SDS of TH comparing the colony types to the previously isolated strains from 30–35 day old BIP/BIN seedlings. Further, different seed lots were cultured in vitro in sucrose-minus or sucrose-plus MS medium and monitored for the immediate or delayed expression of microbial growth, or tested for any covert bacterial associations through medium-indexing. In a subsequent experiment, the different parts of 6 weeks-old BIP seedlings, namely, root, collar tissue, hypocotyl, shoot-tip, and the leaf/petiole were monitored for internal bacterial colonization by placing tissue segments from surface sterilized seedlings on NA. This involved the standard surface sterilization steps as above except for using NaOCl with 1.0% available chlorine in place of 4% chlorine. BIN seedlings were tested without the surface sterilization. Further, different seed lots were tested adopting the standard surface sterilization procedure or with modifications involving reduced or extended disinfection treatments (1% or 4% chlorine for 5/10 min).

### 2.13. Experimental Design and Statistical Analysis

The PGP experiments were laid out in a completely randomized design as earlier [10] employing three replications. The data were subjected to single factor analysis of variance employing the data analysis package of MS Excel 2007 and the mean ± SD values are presented. A benchmark of ≥20% increase in performance over the uninoculated control was adopted as the indicator of beneficial effect by the inoculants.

### 2.14. Accession Numbers

The 16S rRNA sequence data of the distinct bacterial isolates from “Arka Vikas” and “Arka Abha” in vitro seedlings in this study have been deposited with the NCBI database with the accession numbers MK039407 to MK039419.

## 3. Results

### 3.1. Seed Surface Sterilization

The initial wash solutions from both “Arka Vikas” and “Arka Abha” seeds (direct and after Tween-20 step) showed several bacterial colonies on NA, which proved to be mostly Gram-positive spore-forming *Bacillus* spp. (Figure S1). Very few colonies were observed after the ethanol wash and none after the NaOCl treatment. The three seed lots varied to some extent in the initial bacterial cfu, colony morphotypes and the effectiveness of surface sterilization. Normally no microbial survival was observed after the NaOCl treatment. However, in some instances, a few spore-forming bacteria appeared in the wash solutions. In such instances, the seed/seedling cultures tended to show obvious bacterial growth on sucrose-plus medium or covert bacterial association in sucrose-minus MS medium during the culture indexing undertaken after 7–20 days. Such batches were excluded from the downstream analysis on seed endophytic bacterial associations. Fungal colony growth was observed in some instances (5–20% cultures in sucrose-plus medium and 0–10% tubes on sucrose-minus MS). Results from seeds cultured in sucrose-minus MS medium only are considered in this report.

### 3.2. Testing the Seed Homogenate and Germinating Seeds

TH from the three seed lots that were efficiently surface sterilized (without any colony growths from the wash solutions or seed imprints) did not show any bacterial cfu on NA or TSA during the one week of observation. Particulate matter was obvious at 10^0^ sample-applied spots which mimicked colony growth (Figure S2) but yielded no cfu upon its re-streaking or dilution-plating suggesting the absence of any cultivable bacteria associated with the surface sterilized seeds. TH from day-4 germinating seeds on MS medium in culture tubes did not yield any cfu but for the particulate spots at 10^0^ on NA/TSA, re-streaking of which again did not yield any colony growths.

### 3.3. Testing Cotyledonary Stage In Vitro Seedlings

“Arka Vikas” and “Arka Abha” seeds registered 98% and 94% germination, respectively, 7-days after in vitro culture. All the seedlings appeared clean and healthy, and they proved BIN as per day-7 indexing. Day-10 seedlings weighed 39.5 mg and 43.0 mg for “Arka Vikas” and “Arka Abha”, respectively, with green cotyledons and emerging shoot tips. The seed-coat appeared detached and distant from the seedling base, or attached to the tip of one of the cotyledons (Figure S3). Seedling TH did not show any bacterial cfu on NA/TSA except for the grainy particles at 10^0^ sample applied spots with no further colony growths upon re-streaking negating the presence of any cultivable bacteria on two common bacteriological media. The pooled homogenate from the empty-seed coats showed a slow colony outgrowth after 4–7 days. “Arka Vikas” yielded just one colony type from 20 empty seed-coats (identified as *Staphylococcus epidermidis*) while “Arka Abha” exhibited a filamentous *Streptomyces* which failed to revive after the re-streaking.

### 3.4. Testing One Month Old In Vitro Seedlings

**Figure 1:**
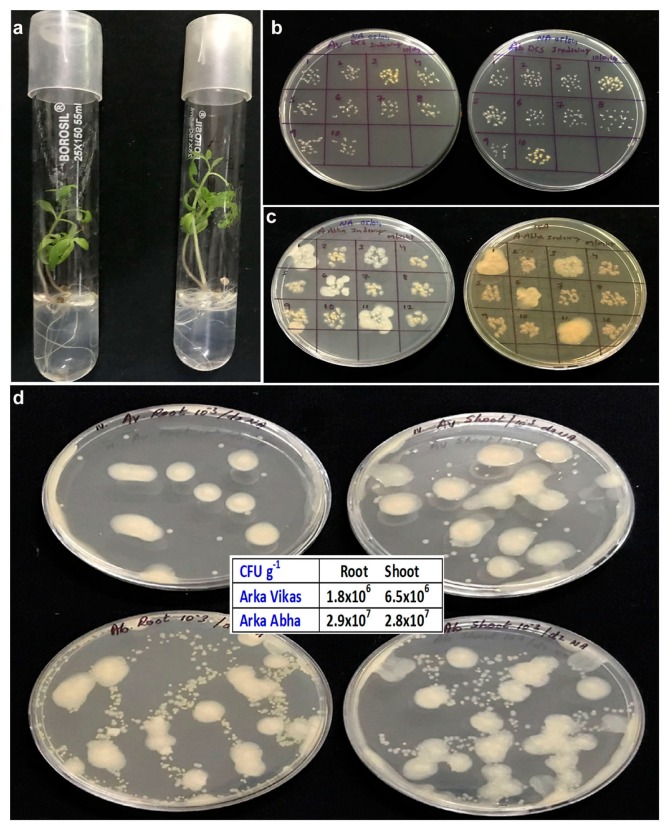
Indexing of visibly clean tomato seedling cultures for bacterial association and the isolation of associated bacteria. (**a**) One month old visibly clean in vitro seedlings of tomato “Arka Vikas” and “Arka Abha”, (**b**) indexing of seedling cultures at two weeks from initiation showing bacterial association in 10–30% cultures on nutrient agar (NA) after overnight incubation, (**c**), indexing of 4 week old “Arka Abha” seedlings on two media (NA and trypticase soy agar (TSA) showing the association of different bacteria 3 days after the indexing, (**d**) bacterial cfu from shoot and root tissues of one-month old “Arka Vikas” and “Arka Abha” seedlings displaying different colony types.

Seedlings appeared clean and healthy excluding about 6–10% that showed fungal growth within 1–2 weeks. The empty seed-coats were removed during the culture indexing undertaken by two weeks. About 20–30% seedlings appeared as BIP after 2 and 3 weeks. By 4 weeks, 25–30% seedlings of “Arka Vikas” and 70–80% seedlings in “Arka Abha” proved BIP while remaining visibly clean or devoid of any obvious bacterial colony growths. The indexed-spots on NA/TSA displayed white, cream or yellow colony types (Figure 1a–c).

### 3.5. Isolation and Identification of Bacteria from BIP Seedlings

“Arka Vikas” seedlings appeared relatively more vigorous compared with “Arka Abha” (average shoot weights of 99.2 and 72.0 mg, and root weight, 59.6 and 33.6 mg, respectively). Root and shoot tissue wash solutions from BIP-seedlings showed “too many colonies to count” in 100 μL sample applied plates. One fast-growing large fluidy white colony type appeared dominating along with numerous underlying small colonies in both the cultivars. 

TH from the root and shoot tissues showed distinct cfu at 10^3^ dilution registering 10^6^–10^7^ cfu g^−1^ with relatively more cfu for “Arka Abha” than “Arka Vikas” and more cfu for shoot tissues than roots in “Arka Vikas” (Figure 1d). The fast-growing white fluidy colony type (identified subsequently as *Enterobacter oryzendophyticus/Kosakonia oryzendophytica*) appeared domineering in both root and shoot tissues of either cultivar. Three other colony types, namely, small white (*Ralstonia pickettii*), yellow (*Sphingomonas paucimobilis*), and cream (*Sphingobium yanoikuyae*) appeared in the background, which became obvious after 2–3 days and at higher dilutions. As per cfu abundance, “Arka Vikas” showed predominantly *R. pickettii* followed by *S. paucimobilis*, *K. oryzendophytica* and *S. yanoikuyae* while “Arka Abha” displayed more of *S. paucimobilis* followed by *R. pickettii*, *K. oryzendophytica* and *S. yanoikuyae*. Some additional colony morphotypes were picked up from “Arka Abha” upon extended incubation for 4–7 days. Four distinct colony types were finally selected from “Arka Vikas” and nine from “Arka Abha” with the above-mentioned four organisms common to both cultivars (Table 1). The other organisms included *Micrococus aloeverea*, *Ralstonia mannililtolilytica* and *Brachybacterium conglomaratum* plus two isolates each of *R. pickettii* and *S. paucimobilis*. The endophytes included α-, β-, and γ- Proteobacteria, and Actinobacteria with the dominance of Gram-negative bacteria (100% in “Arka Vikas” and 78% in “Arka Abha”). SP-SDS of MS culture medium showed 10^6^ cfu g^−1^ for “Arka Vikas” and 10^7^ cfu g^−1^ for “Arka Abha”, with the four major organisms in both the cases, but for some variation in the dominance of different colony morphotypes. The bacterial presence was not at all obvious in the seedling culture medium. No bacterial colony growth was observed with the control MS culture medium.

### 3.6. Isolation and Identification of Bacteria from BIN Seedlings

The second-round testing of BIN seedlings 32-days after the in vitro seed culturing showed consistent results with no normally cultivable bacteria detected in the medium. The wash solutions from the day-35 BIN seedlings also did not display any colony growths on NA/TSA. TH, on the other hand, showed abundant bacterial colonies in “Arka Vikas” with a relatively higher cfu g^−1^ tissue for shoot tissues (4.4 × 10^6^) than roots (3.5 × 10^5^). “Arka Abha” BIN seedlings yielded very few cfu, which too confined to shoot tissue (2 × 10^3^). The two colony morphotypes associated with “Arka Vikas” were identified as *S. paucimobilis* and *Kocuria* sp. with the latter showing 96% homology to *K. subflava* (Table 2). “Arka Abha” isolates included *Micrococus aloeverea* and *Bacillus* sp. (98% homology to *B. phocheonensis*) with the latter proving to be an unusual Gram-negative Firmicute with delayed spore formation. SP-SDS of the culture medium on NA showed 10^4^ cfu g^−1^ MS medium for “Arka Vikas” with the two above isolates while “Arka Abha” culture medium showed no cultivable bacteria.

### 3.7. Growth of Isolates on Differential Media and PGP Characteristics

**Figure 2:**
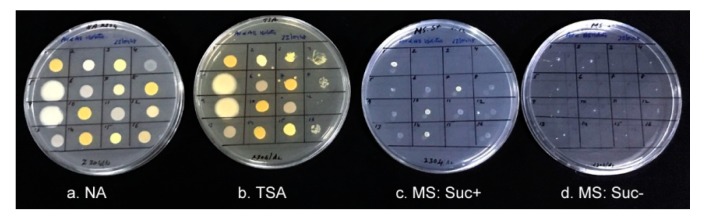
Growth of endophytic bacterial isolates from tomato on four different enriched media four days after inoculation. (**a**) nutrient agar, (**b**) trypticase soy agar, (**c**), sucrose-plus MS medium, and (**d**), sucrose-minus MS medium; c and d gelled with 3 g L^−1^ Phytagel®; 1–4, isolates from index-negative seedlings as per Table 2 and 5–16, isolates from index-positive seedlings as per Table 1 (excluding IPS. Ab09 isolate).

The bacterial isolates from 1-month-old BIP and BIN seedlings showed no detectable colony growths on phytagel-gelled sucrose-minus medium during the two week period of observation but displayed very faint colony growths on sucrose-plus MS medium for most of the isolates. Normal colony growth was observed on control NA and TSA within 1–2 days that served as the evidence for ample bacterial inoculums (Figure 2). Based on the identification results and similarity in growth/colony characteristics on differential media, five distinct bacterial isolates from “Arka Vikas” including four from BIP seedlings and one from BIN seedlings (Tm.Vt-SE.Av01 to Av05) were picked up for detailed studies. Eight isolates were selected from “Arka Abha” (Tm.Vt-SE.Ab01 to Ab08) including seven from BIP seedlings and one from the BIN source (Table 3). Although *K. oryzendophytica* (IPS-Av01 and IPS-Ab01 isolates) displayed fast growth on NA and TSA, they appeared shy to grow on MS (plus or minus sucrose). In agar-gelled MS medium (with or without sucrose) all the isolates except *Micrococus aloevereae* showed detectable growth. The 0.1 OD stocks used in different experiments showed varying cfu depending on the organism providing an insight into the amount of inoculums used in different experiments.

Assessing the PGP characteristics, all the *Ralstonia* isolates and *Brachybacterium* sp. exhibited P-solubilisation capability (Table 3). None of the strains showed the capacity for N-fixation based on growth assessments in Jensen’s liquid medium although most isolates displayed colony growth on agar-gelled Jensen’s medium and on water agar. All the isolates except *Micrococcus* sp. exhibited ammonia production while none showed indole production as the indicator of bacterial auxin activity.

### 3.8. HTE-Based Host Reliance Assay

**Figure 3:**
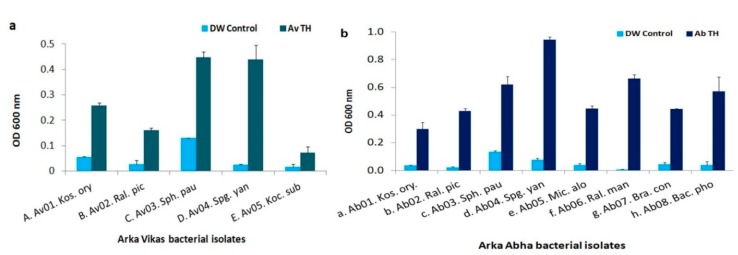
Host tissue extract (HTE)-based host reliance assay on the bacterial endophytes from tomato “Arka Vikas” and “Arka Abha” (as per Table 3) employing MS liquid basal medium. (**a**), “Arka Vikas” seedling isolates in MS liquid medium supplied with 10% (v/v) “Arka Vikas” HTE and (**b**), “Arka Abha” isolates supplied with 10% “Arka Abha” HTE. Growth assessed based on OD_600nm_ 2-days post-inoculation.

The “Av” isolates inoculated in sucrose-minus MS liquid medium showed very slow growth 2-days post-inoculation except for *S. Paucimobilis*, while the “Arka Vikas” HTE supplementation significantly enhanced the growth of all the isolates (Figure 3a) with *S. yanoikuyae* registering the best response (18 folds increase in OD) over the FDW control. The HTE effect appeared the least for *S. paucimobilis* (3.4×) where the organism displayed some growth in the sucrose-minus medium. The trial involving “Arka Abha” HTE on “Ab” isolates also showed similar results with the maximum growth enhancement documented in *S. yanoikuyae* (Figure 3b). The lowest growth response to HTE supplementation was observed for *Kocuria* sp.

### 3.9. Endophyte-Mediated Host Benefits

**Figure 4:**
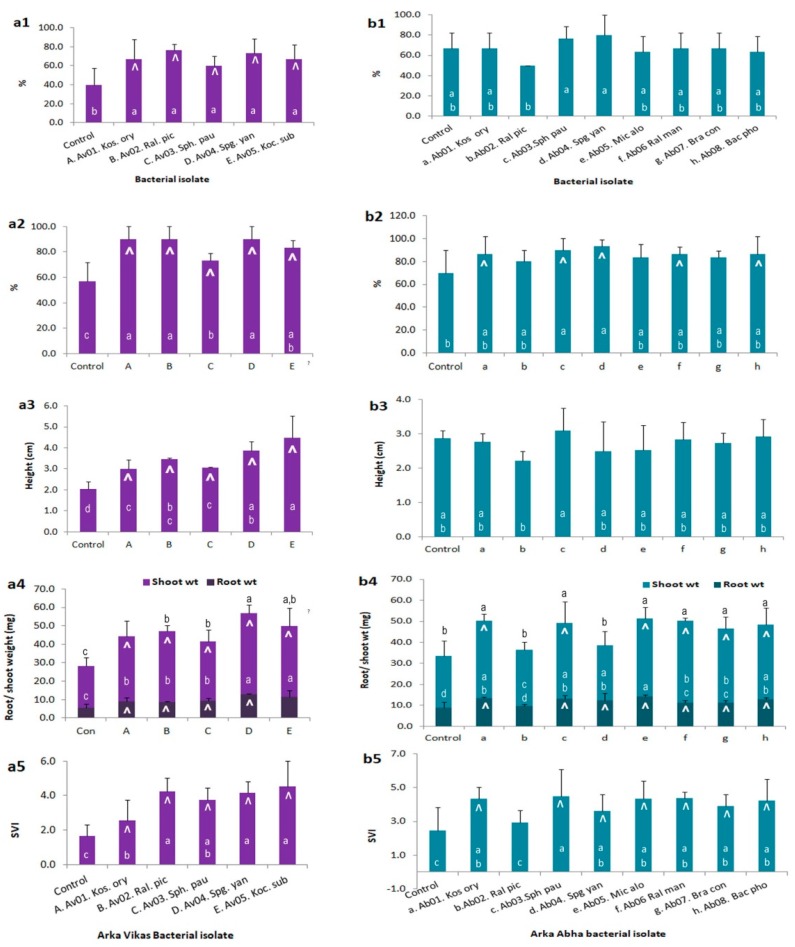
Assessing the endophyte-mediated host beneficial effects through in vitro trials. (**a**) “Arka Vikas” seed inoculation with the “Av” isolates and (**b**) “Arka Abha” seed inoculation with the “Ab” isolates: Series 1, percent germination as on day 7; 2, seedling output after 2 weeks; 3, average seedling height; 4, root/shoot weight per seedling and 5, seedling vigor index (SVI). Upward arrow mark indicates ≥ 20% increase over un-inoculated control. Bars marked with varying letters represent treatments differing significantly from each other.

“Av” isolates displayed beneficial effects in vitro following seed inoculation hastening seed germination in all instances (Figure 4a). Keeping a benchmark of ≥ 20% increase over uninoculated control as the indicator of a notable beneficial effect, all the five isolates displayed higher seedling output, seedling height, root and shoot weights, and SVI in “Arka Vikas” over FDW control after two weeks. The beneficial effect was relatively less pronounced with the “Ab” isolates on “Arka Abha” wherein only five isolates displayed enhanced root and shoot growths (Figure 4b). A significant increase in SVI over control treatment was observed with all the isolates in both the cultivars. Assessing the relative contribution of root and shoot parts to improved seedling vigour, the shoot component contributed relatively more to the higher seedling weight than the root part.

The repeat trial showed *Kosakonia* and *Sphingobium* spp. from “Arka Vikas” as more promising (20 and 21.5% higher SVI over DW control) in “Arka Vikas”. Among the “Ab” isolates on “Arka Abha”, only *Spingomonas* appeared rewarding (10.5% higher SVI over control). A third trial showed *Sphingobium* as a consistent growth enhancing endophyte of “Arka Vikas”. In “Arka Abha”, *Brachybacterium, Micrococus* and *Bacillus* spp. proved more rewarding. Some inconsistency in performance was observed in repeat trials which may be linked to the activation of native endophytes as indicated below.

### 3.10. Testing the Re-Colonization Efficacy of Endophytic Bacterial Isolates

**Figure 5:**
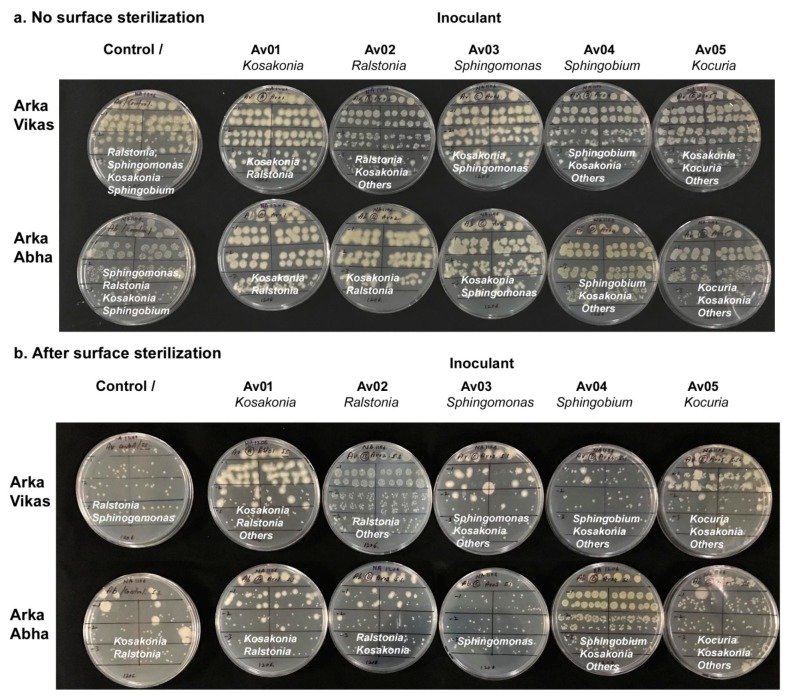
Testing the re-colonization efficacy of endophytic bacterial isolates through seed soaking inoculation of surface sterilized seeds of tomato “Arka Vikas” with single “Arka Vikas” isolates Tm-Vt-SE-Av01 to Av05. (**a**) Monitoring the root tissues of in vitro seedlings after two weeks of seed inoculation without surface sterilization, and (**b**) after surface sterilization using NaOCl, displaying the association of multiple organisms in uninoculated control and the inoculant plus additional organisms in the seed inoculated set.

“Arka Vikas” and “Arka Abha” seed inoculation with the “Av” isolates resulted in bacterial cfu of 10^8^–10^9^ g^−1^ root tissues in two-week-old seedlings that constituted the inoculated organism plus one or more additional organism(s) (Figure 5a). Despite the absence of any colony growths during the monitoring of seed-wash solutions, the control sets in both “Arka Vikas” and “Arka Abha” displayed bacterial association (10^6^–10^8^ cfu g^−1^ tissue). Based on colony characteristics, the bacterial associates from “Arka Vikas” control set appeared to be a mixture of *Ralstonia pickettii* followed by *Sphingobium*, *Kosakonia*, and *Sphingomonas* spp. The control seedlings in “Arka Abha” showed predominantly *Sphingomonas* followed by *Ralstonia* and occasional *Kosakonia* and *Sphingobium* spp. *Kosakonia* inoculated “Arka Vikas” showed largely *Kosakonia* with underlying *Ralstonia*; seedlings derived after *Sphingomonas* inoculation also had a dominance of *Kosakonia*. Seeds inoculated with *Ralstonia*, *Sphingobium*, and *Kocuria* spp. showed mostly the inoculated organism in the seedlings with accompanying *Kosakonia*. Some additional colony morphotypes were also observed in some treatments, indicating the activation of different bacteria from uncultivable state to cultivation considering that the wash solutions or the seed homogenate from surface sterilized seeds did not show any cultivable organisms at seed inoculation.

Following surface sterilization, the control sets in both “Arka Vikas” and “Arka Abha” (Figure 5b) showed the association of two or more colony types suggesting the activation of different species or the multiplication of lowly associated organisms during the two weeks of in vitro phase. Tm-Vt-SE-Av01 to Av05 seed-inoculated sets showed recolonization by the respective organism together with the association of one or more additional organisms. Activation of *Kosakonia* was obvious in “Arka Vikas” seedlings derived after inoculation with *Sphinogomonas*, *Sphingobium*, and *Kocuria* spp. In “Arka Abha”, *Ralstonia* inoculation activated *Kosakonia* while in other instances, no notable additional organism was observed.

### 3.11. Monitoring In Vitro Cultures at Different Phases of Growth and In Vitro Monitoring of Different Seed Lots

In this trial involving 6-month-old seeds in storage, “Arka Vikas” and “Arka Abha” seeds showed 98 and 92% germination, respectively, in MS medium by day-7 with all seedlings appearing clean. Indexing after two weeks showed 100% “Arka Vikas” seedlings as BIN while 4% in “Arka Abha” proved BIP. Indexing after 3 weeks indicated 16% seedlings in “Arka Vikas” and an additional 14% in “Arka Abha” as BIP while that after 4 weeks added another 14% and 16% cultures of “Arka Vikas” and “Arka Abha”, respectively, to the BIP category (final figure 30 and 34%, respectively). Plating the root tissue homogenate from individual seedling showed 10^6^–10^7^ cfu g^−1^ tissue in BIP seedlings with a single organism in most instances unlike the earlier observation employing pooled TH from BIP and BIN older seedlings (Figure S4). The same appeared during the SP-SDS of the medium too with the exclusivity or the dominance of one colony type. Fungal contaminants appeared occasionally and such cultures were excluded from indexing.

Observations six weeks into culturing showed fungal growth in 4/24 of remaining seedlings of “Arka Vikas” and 4/20 of “Arka Abha”. Indexing of the left over cultures indicated 10% seedlings from “Arka Vikas” and 25% of “Arka Abha” as bacteria-harboring. The BIP seedlings in both instances appeared more vigorous compared with the BIN seedlings with a relatively higher shoot and gross seedlings weights (data not presented), but this observation was based on a limited number of saplings and hence inconclusive. BIP seedlings at 7-weeks stage showed 1.8 × 10^6^ to 2.9 × 10^7^ cfu g^−1^ root tissue with the association of different organisms.

**Figure 6:**
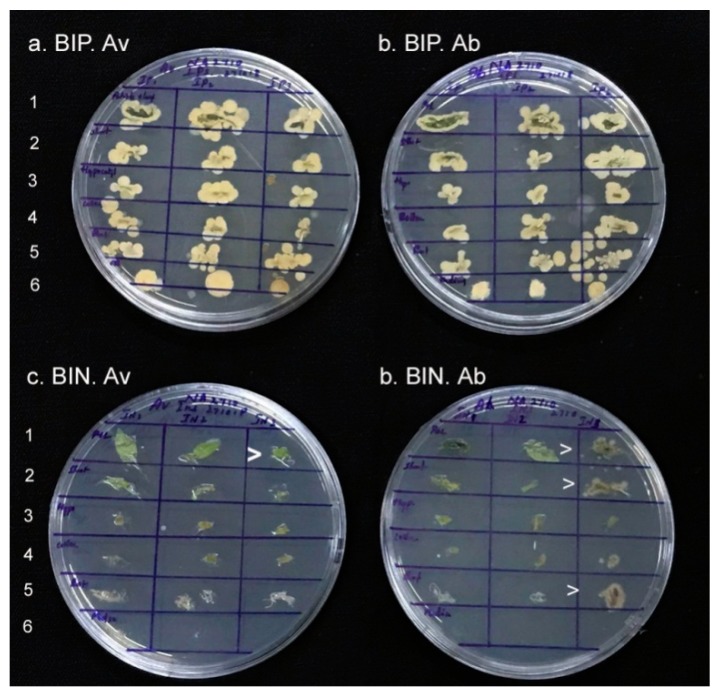
Monitoring the tissue parts from the different parts of index-positive (BIP) seedlings and index-negative (BIN) 6 week old in vitro seedlings of tomato. (**a**,**b**) surface sterilized BIP seedlings of “Arka Vikas” and “Arka Abha”, respectively, and (**c**,**d**) BIN seedlings tested directly. 1–6 stands for leaf/petiole, shoot tip, upper hypocotyl, collar region, roots and growth medium, in that order, and the arrow-head denotes bacterial growth from BIN seedlings.

In the next experiment where the BIP seedlings were monitored employing tissue segments from surface sterilized seedlings, the bacterial association was observed in all tested parts, namely, the roots, collar region, hypocotyl, shoot-tip, and the leaf/petiole (Figure 6). The shoot-tip and leaves displayed relatively more bacterial colony growths than roots despite the tissues getting partly bleached. The bacterial detection in the medium endorsed the passage of endophytes from the plant/roots to the rhizosphere. Seedlings those were BIN during medium-indexing showed bacterial colonization in localized pockets with no cultivable bacteria detected in the medium.

Repeat trials involving different seed lots where the seeds were cultured singly on MS medium in glass tubes showed some lot-to-lot or batch-to-batch variations in the extent of BIP seedlings after 1–4 weeks. A number of additional bacteria were found to be associated with the in vitro seedling cultures the extent of which varied with the seed lot and the experimental batch. These included *Enterobacter*, *Pseudomonas*, *Acinetobacter*, *Micrococcus*, *Paenibacillus*, and *Bacillus* spp. Some seed lots and surface sterilized batches showed a few bacterial colonies in the wash solution after the NaOCl treatment (Figure S5). This appeared either due to a difference in the initial bacterial load or a reduction in the efficacy of the NaOCl lot after the initial opening the container. In such instances, more in vitro seedlings proved index-positive gradually. Compared to the surface sterilized seeds planted individually in MS-based culture tubes, bulk seeds incubated together in culture plates frequently showed BIP status. This appeared to be contributed by the lateral spread or multiplication of any bacterial escapes, or the activation of uncultivable bacteria.

## 4. Discussion

Plants have been normally considered to acquire endophytic microorganisms from their surroundings mainly at seed germination and early seedling growth, primarily from the spermosphere and the rhizosphere [2,3,12]. Other possible routes of bacterial entry include through stomata, floral parts, natural openings, wounds, and through insect vectors and pollinators, while vertical transmission through seeds has also been documented [2,4,12]. Results from the present investigations on tomato revealed the seed internal association and embryo-transmission of different endophytic bacteria and their integration with the growing seedlings. Seed colonization by endophytic bacteria has been documented in other cultivation-based and cultivation-independent studies [8,33], also validated through microscopy in some instances [17,34]. Further, the pollen-mediated transmission of endophytes has been documented in some plant species [4,35] which reinforces their vertical transmission. In wheat it has been possible to drive in selected bacteria inside seeds by spraying the bacterial suspension at flowering [36].

Several bacteria are described as viable-but-non-cultivable (VBNC) [37], or viable-but-not-yet-cultivable (VBNYC) in that their growth requirements are yet to be understood [38]. The non-recovery of cultivable organisms from surface-sterilized seeds, germinating seeds or cotyledonary stage seedlings on common enriched media in this study indicated that the allied organisms were largely VBNC, or surviving in a dormant state and hence normally difficult to isolate [8]. The gradual transition of a share of seedlings in sucrose-minus MS medium from BIN state to BIP status appeared attributable to the activation of VBNC bacterial cells to normal cultivable form with the seedling growth. This activation effect was not merely due to the imbibing of moisture or seed germination. The bacterial emergence also appeared unlikely due to the cells coming in contact with the culture medium which option was available while applying the TH directly on enriched media. It may be noted that all the organisms reported here are normally cultivable. Large shares of endophytic bacteria are known to be not amenable to cultivation [2,3,4,6,27], but once brought to cultivation, many appear to be normally cultivable organisms [26,38]. Plant tissue cultures often show bacterial contamination with culture aging or upon sub-culturing of older/senescent stocks, possibly arising from the activation of VBNC cells elicited by the release of tissue breakdown products, pH changes and other factors [25,26,31]. The use of specialized media or the supplementation of medium with the HTE could trigger the conventionally uncultivable bacteria in banana/watermelon to cultivation [26,38]. Further, a number of bacteria were induced to cultivation when the seeds were kept together in the Petri-dishes or post-inoculation with single organisms. A better terminology to describe such endophytes could be ‘cultivation recalcitrant endophytic bacteria’ (CREB) as documented through cultivation-independent molecular approaches in different crops [39,40].

Observations with the one-month-old BIP seedlings showed the dominance of *Ralstonia pickettii* in “Arka Vikas” followed by *Sphingomonas paucimobilis*, *Kosakonia oryzendophytica*, and *Sphingobium yanoikuyae*. “Arka Abha” also showed the same four organisms but differed in the abundance rankings. In both the cases, Proteobacteria formed the dominant phylum followed by Actinobacteria with the majority constituting Gram-negative bacteria consistent with the general observations on bacterial endophytes [1,3]. *K. oryzendophytica* was first isolated as *Enterobacter oryzendophyticus* as a nitrogen-fixing and phosphate supplying rice root endophyte [41], and subsequently reclassified as *Kosakonia* sp. [32]. This organism appeared as an intensely seed-associated endophyte in tomato being observed with different seedling batches. *R. pickettii* has been isolated as a root and seed endophyte in tomato, known for plant growth promotion and pathogen antagonism [8]. *Sphingomonas paucimobilis* has been isolated as a growth-promoting endophyte in Dendrobium [42]. *Sphingobium yanoikuyae*, previously known as *Sphingomonas yanoikuyae* or *Beijerinckia* sp. known for its versatile capabilities to degrade soil pollutants [43] is now observed as a seed endophyte. Other less abundant organisms isolated from “Arka Abha” seedlings have been reported previously as endophytes, such as *Micrococus aloevereae* in *Aloe vera* [44] and *Brachybacterium conglomaratum* in tea [45].

Gram-positive *Bacillus* spp. and the related spore-forming genus of *Paenibacillus* formed the common seed-associated endophytes isolated in different plant species [12,13,46] including tomato [14,15] as per cultivation-based assessments on surface sterilized seeds. This is possible considering the non-exclusion of seed coat and the long dry spell the seeds are exposed to during the processing and in storage. A cultivation-based study of bacterial endophytes within surface sterilized seeds of two tomato hybrids showed a bias towards spore-forming bacteria (*Bacillus*, *Paenibacillus*, *Jeotgalibacillus*, and *Psychrobacillus* spp.) with lower shares of other bacteria while the16S rRNA gene amplicon-based study showed considerable diversity under the phyla Firmicutes, Proteobacteria, Actinobacteria, and Bacteroidetes with no significant match to the cultivation-based study [15]. Thus, a variation in microbial flora was observed between cultivation-based and molecular approaches besides the genotypic effects. The cultivation-based experimental method in our study was similar in approach to that of isolating bacteria from axenically grown seedlings in the cited study [15], but for the care taken to exclude the seed coat tissues. The outcome was the recovery of predominantly Gram-negative bacteria (88% isolates). Only one member of Firmicutes was isolated with 98% 16S rRNA gene sequence homology to *Bacillus phocheonensis* which in turn proved to be a rare Gram-negative delayed spore-former. In our experience, *Bacillus* spp. were associated with the exterior of dry seeds, frequently retrieved from seed wash solutions and as contaminants in axenically raised seedlings on sucrose-plus MS medium. Studies on seed-associated bacteria in tomato relied on surface sterilized seeds which did not exclude the seed coat tissues [8,14,15]. Seed transmission of *Bacillus* spp. perhaps depends on external association to the embryonic tissues rather than embryo transmission per se. It warrants a molecular analysis on embryonic tissues to assess the vertical transmission of *Bacillus* sp. and related genera of spore-formers.

The observation that several Gram-negative bacteria being capable of surviving in dry seeds in a low moisture environment that were in storage for >6 months was significant. Gram-negative genera such as *Pseudomonas*, *Enterobacter*, *Pantoea*, *Acinetobacter*, and non-spore forming Gram-positive *Micrococcus*, and *Staphylococcus* have also been found to colonize seeds [12,13,47,48]. In rice Gram-negative isolates predominated in the initial stages of seed development and Gram-positive isolates appeared as the seeds matured [47]. A number of additional bacteria than those described in this study were found to be associated with the in vitro seedling cultures the extent of which varied with the seed lot and the experimental batch. This study was not aimed at gathering all such seed colonizing bacteria but just proving the seed endophytic association utilizing the in vitro experimental system. It warrants detailed cultivation-independent molecular analyses on seed embryonic tissues to confirm the true vertically transmitted bacteria. In either case, the seed-associated organisms generally get the benefit of early seedling colonization at germination [4,12,13]. As of now, there are about 155 bacterial genera detected inside seed tissues of various host plants [20].

The tissue culture system proved to be a very useful tool to study the seed-associated bacteria away from other intruding/external organisms as opposed to the soil-grown plants [7,26]. The in vitro system also offered the scope to assess the host–endophyte mutualistic or symbiotic effects with the use of single organisms. This might be different from the natural conditions but in fact a more accurate indication of the capability of the individual organisms. The HTE-mediated growth enhancement of bacterial isolates indicated that the endophytes derived a beneficial effect from the host associations. Considering the host-benefits from the association, most of the isolates contributed to early seed germination, better seedling output or seedling vigour under in vitro conditions. This is despite the organisms not showing the normally acclaimed PGP indicators except for the P-solubilisation characteristic of *Ralstonia* and *Brachybacterium* spp. This suggested the involvement of other contributing factors beyond those covered in this study such as the effect due to the rhizophagy cycle [19]. All the isolates except *Micrococcus* sp. showed ammonia production which characteristic is often associated with tissue decaying microorganisms [49]. While all the organisms possibly benefitted from the host association, the benefit to the plant varied with the organisms. Under natural conditions, this may be additionally influenced by a series of other factors. It was significant to note a relatively higher cfu for the shoot tissues compared with the roots or the seedling growth medium contrary to the general observation with the soil-grown plants. The bacterial presence in the medium also indicated the passage of endophytic bacteria from roots to the rhizosphere which has been documented in other studies [48,50]. It may also be noted that the growth advantage for the seedlings following seed inoculation was reflected more with shoots than root growth.

An earlier study involving tomato cultivars that differed in the reaction to the bacterial wilt pathogen *Ralstonia solanacearum*, including four resistant and four susceptible cultivars, showed higher endophytic bacterial diversity and more organisms bearing antagonistic potential against the pathogen in resistant than susceptible genotypes [51]. In the present study too, “Arka Abha” which is resistant to *R. solanacearum* showed more bacterial diversity compared with the susceptible ‘Arka Vikas. Assessing the biocontrol potential of these organisms did not form a part of this study. As for the practical exploitation of the identified organisms in agriculture, more realistic assessments under natural conditions will be required to appreciate the potential agronomic applications. Further, the ability of the introduced bacteria to survive on the seeds or soil and to colonize the seedlings is influenced by soil and edaphic factors besides the micro-microbe interactions [52]. The present study was aimed only at the elucidation of the seed association of endophytic bacteria and to understand the nature of the relationship. The observations confirmed the seed internal association of endophytic bacteria and their transmission to the seedlings in line with the recent observations on bacterial transmission between the two successive generations in tomato [8].

In conclusion, this study has brought to light the integral internal seed bacterial association describing some of the dynamics in the relationship between seed-transmitted endophytic bacteria and tomato plants. The dry seeds while harboring an array of external organisms, predominantly spore-formers, also harbored endophytic bacteria internally in a cultivation recalcitrant form. Post-germination under the axenic environment, a share of CREBs showed the activation to cultivable form with the gradual passage of organisms to the rhizosphere. Thus the seeds proved to be packaged with an array of endophytic bacteria that could move internally to various plant organs with the seedling growth. The activated endophytes in controlled studies shared a mutualistic association with the host. In vitro system proved to be a valuable tool to study the endophytic microorganisms protected from external organisms, to study the bacterial activation and to assess the mutualistic effects between the endophytes and the host. NGS studies on exclusive embryonic tissues are warranted to get a full proof picture of vertical transmission of endophytes, and further glasshouse/field studies on PGP and biocontrol potential for a realistic assessment of the isolated organisms for practical exploitation.

## Figures and Tables

**Table 1:** Identification of bacterial strains isolated from 30-day-old in vitro grown bacteria index-positive seedlings of tomato “Arka Vikas” and “Arka Abha”.

S. No.	Isolate	16S seq. (bp)	Closest NCBI Type Strain ^†^	Closest RDP Match and Score ^†^	Suggested ID and Taxonomic Class	Gram Reaction
Isolates from Arka Vikas						
01	IPS. Av01	820	*Enterobacter oryzendophyticus* (NR.125586; 99%)	*Kosakonia arachidis* (S002151030; 0.921)	*Kosakonia oryzendophytica*^Ψ^; γ- Proteobacteria	-ve
02	IPS. Av02	810	*Ralstonia pickettii* (LN681565; 99%)	*Ralstonia pickettii* (S000425929; 0.947)	*Ralstonia pickettii*; β- Proteobacteria	-ve
03	IPS. Av03	790	*Sphingomonas paucimobilis* (LN681566; 100%)	*Sphingomonas paucimobilis* (S000437516; 0.993)	*Sphingomonas paucimobilis*; α- Proteobacteria	-ve
04	IPS. Av04	802	*Sphingobium yanoikuyae* (LT899948; 99%)	*Sphingobium yanoikuyae* (S000413467; 0.914)	*Sphingobium yanoikuyae*; α- Proteobacteria	-ve
Isolates from Arka Abha						
01	IPS. Ab01	820	*Enterobacter oryzendophyticus* (NR.125586; 99%)	*Kosakonia arachidis* (S002151030; 0.906)	*Kosakonia oryzendophytica*^Ψ^; γ- Proteobacteria	-ve
02	IPS. Ab02	800	*Sphingomonas paucimobilis* (NR118806; 100%)	*Sphingomonas paucimobilis* (S000437516; 0.983)	*Sphingomonas paucimobilis*; α- Proteobacteria	-ve
03	IPS. Ab03	805	*Ralstonia pickettii* (NR_043152; 99%)	*Ralstonia pickettii* (S000425929; 0.933)	*Ralstonia pickettii*; β- Proteobacteria	-ve
04	IPS. Ab04	790	*Sphingobium yanoikuyae* (LT899948; 99%)	*Sphingobium yanoikuyae* (S000413467; 0.939)	*Sphingobium yanoikuyae*; α- Proteobacteria	-ve
05	IPS. Ab05	745	*Ralstonia pickettii* (LN681565; 99%)	*Ralstonia pickettii* (S000425929; 0.933)	*Ralstonia pickettii*; β- Proteobacteria	-ve
06	IPS. Ab06	730	*Sphingomonas paucimobilis* (LN681566; 100%)	*Sphingomonas paucimobilis* (S000437516; 0.986)	*Sphingomonas paucimobilis*; α- Proteobacteria	-ve
07	IPS. Ab07	840	*Micrococcus aloeverae* (NR. 134088; 100%)	*Micrococcus aloeverae* (S004054721; 0.996)	*Micrococcus aloeverae*; Actinobacteria	+ve
08	IPS. Ab08	840	*Ralstonia mannitolilytica* (NR.025385; 99%)	*Ralstonia pickettii* (S000425929; 0.948)	*Ralstonia mannitolilytica*; β- Proteobacteria	-ve
09	IPS. Ab09	840	*Brachybacterium conglomeratum* (NR.104689; 99%)	*Brachybacterium paraconglomeratum* (S000446670; 0.971)	*Brachybacterium conglomeratum*; Actinobacteria	+ve

NCBI, National Centre for Biotechnology Information; RDP, The Ribosomal Database Project ^†^ as on 10 Oct. 2018; ^Ψ^ as per Li et al. (2016) [32].

**Table 2:** Identification of bacterial strains isolated from 35-day-old in vitro grown bacteria index-negative seedlings of tomato “Arka Vikas” and “Arka Abha”.

S. No.	Isolate	16S seq. (bp)	Closest NCBI Type Strain ^†^	Closest RDP Type Strain and Homology ^†^	Suggested ID and Taxonomic Class	Gram Reaction
Isolates from Arka Vikas						
01	INS. Av. 01	795	*Sphingomonas paucimobilis* (LN681566; 100%)	*Sphingomonas paucimobilis* (S000437516; 0.989)	*Sphingomonas paucimobilis*; α- Proteobacteria	-ve
02	INS. Av. 02	850	*Kocuria subflava* (NR 144586; 96%)	*Kocuria polaris* (S000130611; 0.856)	*Kocuria* sp.; Actinobacteria	+ve
Isolates from Arka Abha						
01	INS. Ab. 01	815	*Micrococus aloevereae* (NR. 134088; 100%)	*Micrococcus aloeverae* (S004054721; 0.990)	*Micrococus aloevereae*; Actinobacteria	+ve
02	INS. Ab. 02	840	*Bacillus phocheonensis* (NR.014377; 98%)	*Bacillus niacini* (S000014510; 0.879)	*Bacillus* sp.; Firmicutes	-ve

NCBI, National Centre for Biotechnology Information; RDP, The Ribosomal Database Project; ^†^ as on 10 Oct. 2018.

**Table 3:** Characterization of selected seed derived endophytic bacterial isolates from bacteria index-positive (BIP) and bacteria index-negative (BIN) one-month old in vitro seedlings of tomato “Arka Vikas” and “Arka Abha” for colony growth and Plant Growth Promotion (PGP) characteristics.

Isolate Code	Isolate ID	Identity of the Organism	Original Code	Colony Growth/Characteristics					Cell Morphology (d2- NA)	CFU mL^−1^ of 0.1 OD NA Culture	PGP characteristics			
				MSP S^−^	MSP S^+^	MSA S^−^	MSA S^+^	NA/TSA			N-Fixation	Ammonia Production	P-Solubil-Ization	Indole Production
Isolates from Arka Vikas BIP and BIN seedling sources (NCBI 16S rRNA gene accession numbers MK039407 to MK039411)														
A	Tm.Vt-SE.Av01	*Kosakonia oryzendophytica*	IPS. Av 01	-	+	++	+++	+++++; Fast growing fluidy white	Medium size rods	6.50 × 10^7^	-	++	-	-
B	Tm.Vt-SE.Av02	*Ralstonia pickettii*	IPS. Av 02	-	+	++	++	+++; White, medium growth	Small rods	1.50 × 10^7^	-	++	+	-
C	Tm.Vt-SE.Av03	*Sphingomonas paucimobilis*	IPS. Av 03	-	++	++	++	+++; Yellow, medium growth	Small rods	3.10 × 10^8^	-	+++	-	-
D	Tm.Vt-SE.Av04	*Sphingobium yanoikuyae*	IPS. Av 04	-	+	+	++	+++; Light yellow; medium growth	Small rods	1.35 × 10^8^	-	++	-	-
E	Tm.Vt-SE.Av05	*Kocuria subflava*	INS. Av 02	-	-	+	++	+++; Slow white growth	Cocci	7.5 × 10^6^	-	+++		
Isolates from Arka Abha BIP and BIN seedling sources (NCBI 16S rRNA gene accession numbers MK039412 to MK039419)														
a	Tm.Vt-SE.Ab01	*Kosakonia oryzendophytica*	IPS. Ab.01	-	+	+	+++	+++++; Fast growing fluidy white	Medium size rods	9.50 × 10^7^	-	++	-	-
b	Tm.Vt-SE.Ab02	*Ralstonia pickettii*	IPS. Ab.03	-	+	+	++	+++; White, medium growth	Small rods	2.50 × 10^8^	-	+	+	-
c	Tm.Vt-SE.Ab03	*Sphingomonas paucimobilis*	IPS. Ab.02	-	++	+	++	+++; Yellow, medium growth	Small rods	2.10 × 10^8^	-	++	-	-
d	Tm.Vt-E.Ab04	*Sphingobium yanoikuyae*	IPS. Ab.04	-	+	+	++	+++; Light yellow; medium growth	Small rods	1.45 × 10^8^	-	+++	-	-
e	Tm.Vt-SE.Ab05	*Micrococus aloeverae*	IPS. Ab.07	-	+	-	++	+++; Yellow; Slow growth	Cocci	-	-	-	-	-
f	Tm.Vt-SE.Ab06	*Ralstonia mannililtolilytica*	IPS. Ab.08	-	+	+	++	+++; Cream, medium growth	Medium rods	2.45 × 10^8^	-	++	+	-
g	Tm.Vt-SE.Ab07	*Brachybacterium conglomeratum*	IPS. Ab.09		-	-	++	+++’ Light yellow; slow growth	Cocci	1.5 × 10^8^	-	-	+	-
h	Tm.Vt-SE.Ab08	*Bacillus* sp.	INS. Ab.02		-	+	+	++; Light cream flat; slow growth	Medium long rods	8.50 × 10^7^	-	-	-	-

MSP: Murashige and Skoog (MS, 1962) basal medium gelled with Phytagel® at 3 g L^−1^; MSA: MS medium gelled with bacteriological agar at 8 g L^−1^; NA, nutrient agar; TSA, trypticase soy agar. -, nil; +, low; ++, medium; +++, high; ++++, very high.

## Supplementary Materials

The following are available online at https://www.mdpi.com/2076-2607/7/5/132/s1, Figure S1. Monitoring the tomato seeds for the efficacy of surface sterilization through different steps. (a, b) Seed wash solutions of tomato “Arka Vikas” and “Arka Abha” (direct and after Tween-20 step) showing several bacterial colonies on nutrient agar (NA) comprising of *Bacillus* spp./spore formers, and (c, d) very few or no colonies after ethanol wash or NaOCl treatment. Figure S2. Tissue homogenate (100 seeds 10 mL^−1^) from surface-sterilized seeds of tomato “Arka Vikas” and “Arka Abha” and the five decimal serial dilutions applied on nutrient agar showing particulate matter at the original homogenate applied spots (10^0^) mimicking colony growth (with no cfu upon its re-streaking or dilution-plating). Figure S3. Day-10 in vitro grown seedling of tomato “Arka Vikas” and “Arka Abha” on sucrose-minus Murashige and Skoog medium [29] showing clear medium devoid of any microbial association and the seed coat (indicated by arrowhead) detached from the seedling base or carried at the distal end of the cotyledon. Figure S4. SP-SDS on the root tissue homogenate from individual bacteria index-positive 3 weeks old “Arka Vikas” (Av) and Arka Abha (Ab) seedlings showing the association of a single organism in most cases. Figure S5. A different seed lot of the tomato “Arka Vikas” and “Arka Abha” monitored at surface sterilization through seed wash solution spotting on NA (direct and after Tween-20 step) displaying *Bacillus* spp./spore formers (a, b), and inoculum carry over after ethanol wash or NaOCl treatments (c, d) due to difference in the initial bacterial load, or a reduction in the efficacy of the NaOCl.

## Abbreviations

ADW	Autoclaved distilled water
BIN	Bacteria index-negative
BIP	Bacteria index-positive
CREB	Cultivation recalcitrant endophytic bacteria
FDW	Filter-sterilized ADW
HTE	Host tissue extract
MS medium	Murashige and Skoog medium
NA	Nutrient agar
PGP	Plant growth promotion
SATS	Spotting-and-tilt-spreading
SP-SDS	Single plate-serial dilution spotting
SVI	Seedling vigour index
TH	Tissue homogenate
TSA	Trypticase soy agar
VBNC	Viable but non-cultivable
VBNYC	Viable-but-not-yet-cultivable
