# Supplementary material for: In Vitro Activation of Seed-Transmitted Cultivation-Recalcitrant Endophytic Bacteria in Tomato and Host–Endophyte Mutualism
Source: Microorganisms. 2019 May 14;7(5):132. doi: 10.3390/microorganisms7050132 (PMC6560416; doi:10.3390/microorganisms7050132)
Supplement: Supplementary file 1 [file microorganisms-07-00132-s001.pdf]

Supplementary Materials

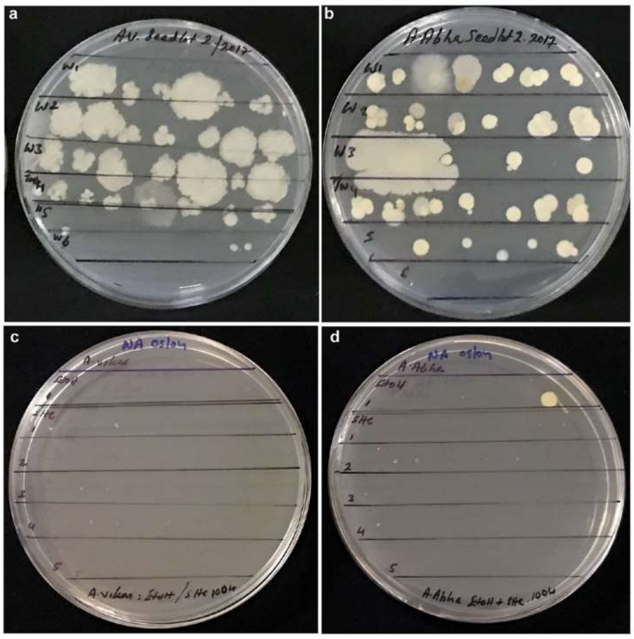

Figure 1. Monitoring the tomato seeds for the efficacy of surface sterilization through different steps. (a, b) Seed wash solutions of tomato 'Arka Vikas' and 'Arka Abha' (direct and after Tween-20 step) showing several bacterial colonies on nutrient agar (NA) comprising of Bacillus spp./ spore formers, and (c, d) very few or no colonies after ethanol wash or NaOCl treatment.

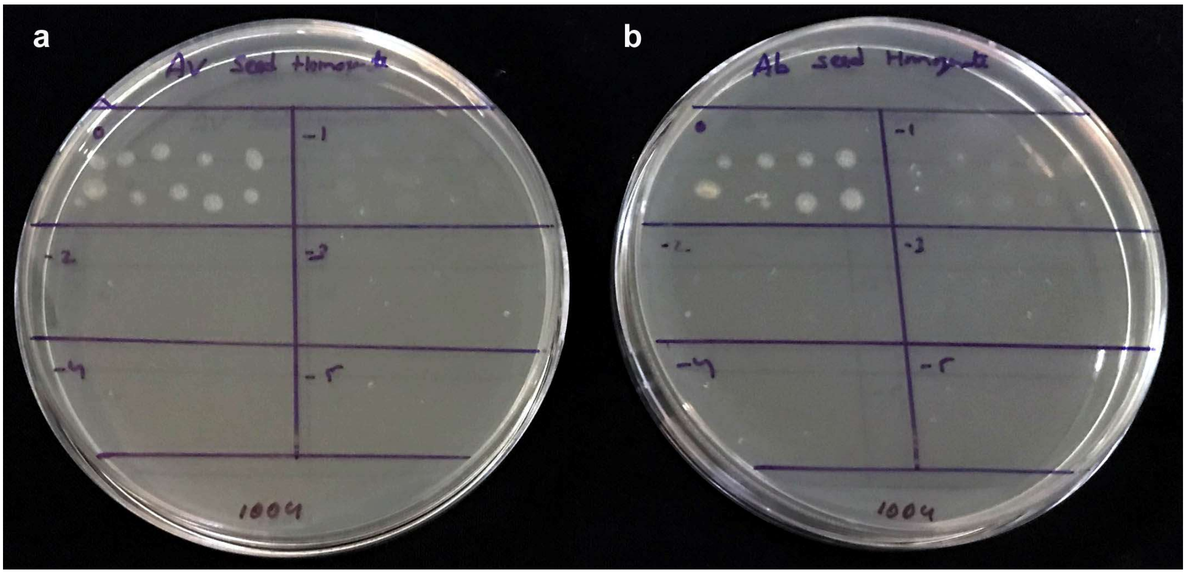

Figure 2. Tissue homogenate (100 seeds 10 ml^{-1}) from surface-sterilized seeds of tomato 'Arka Vikas' and 'Arka Abha' and the five decimal serial dilutions applied on nutrient agar showing particulate matter at the original homogenate applied spots (10^0) mimicking colony growth (with no cfu upon its re-streaking or dilution-plating).

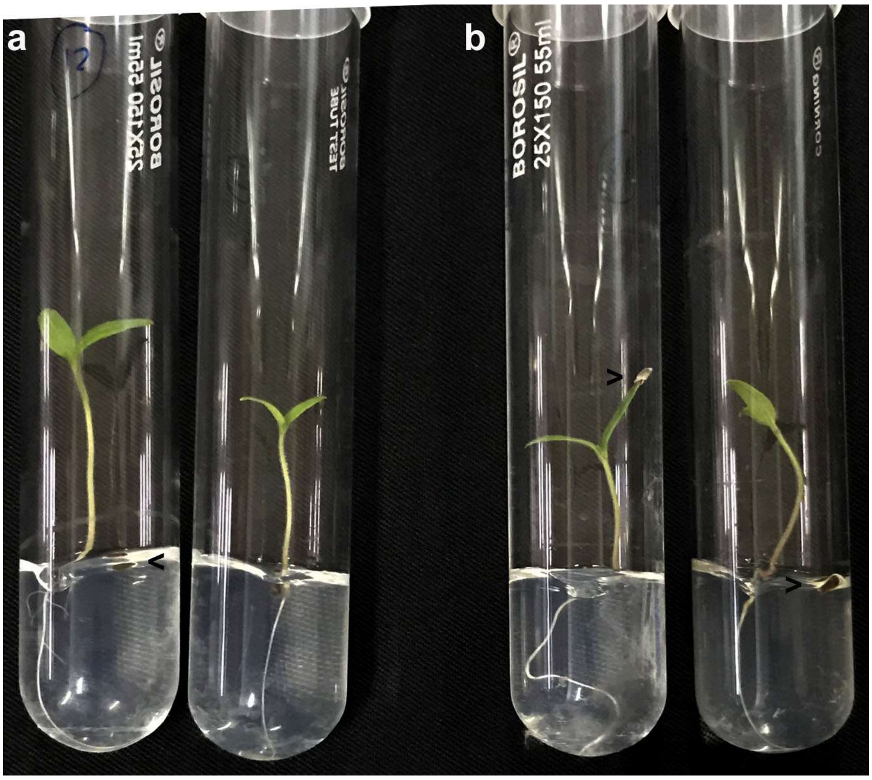

Figure 3. Day-10 in vitro grown seedling of tomato 'Arka Vikas' and 'Arka Abha' on sucrose-minus Murashige and Skoog medium [29] showing clear medium devoid of any microbial association and the seed coat (indicated by arrowhead) detached from the seedling base or carried at the distal end of the cotyledon.

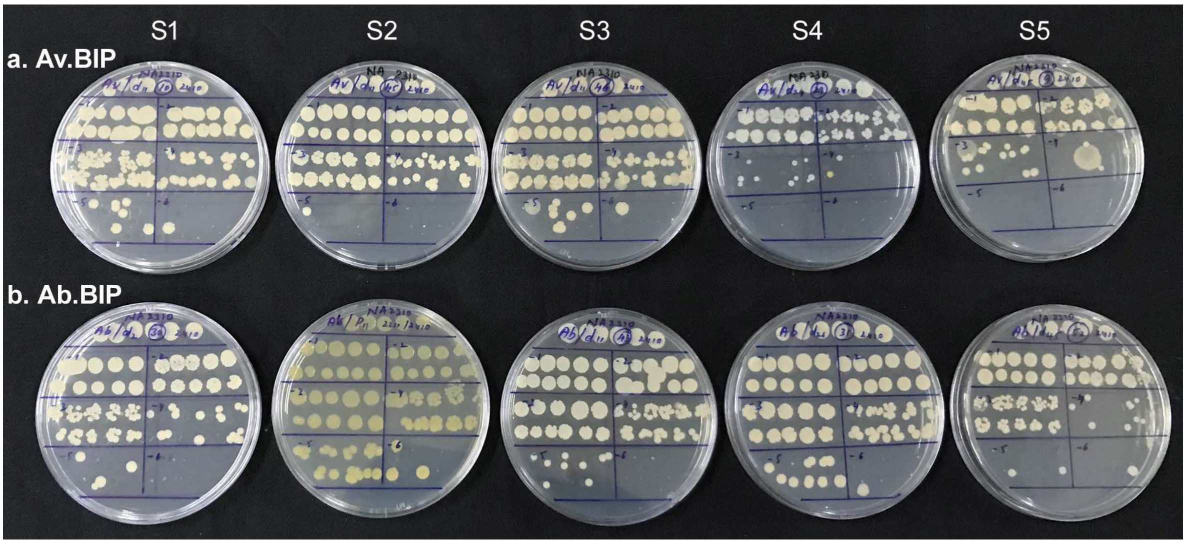

Figure 4. SP-SDS on the root tissue homogenate from individual bacteria index-positive 3 weeks old 'Arka Vikas' (Av) and Arka Abha (Ab) seedlings showing the association of a single organism in most cases.

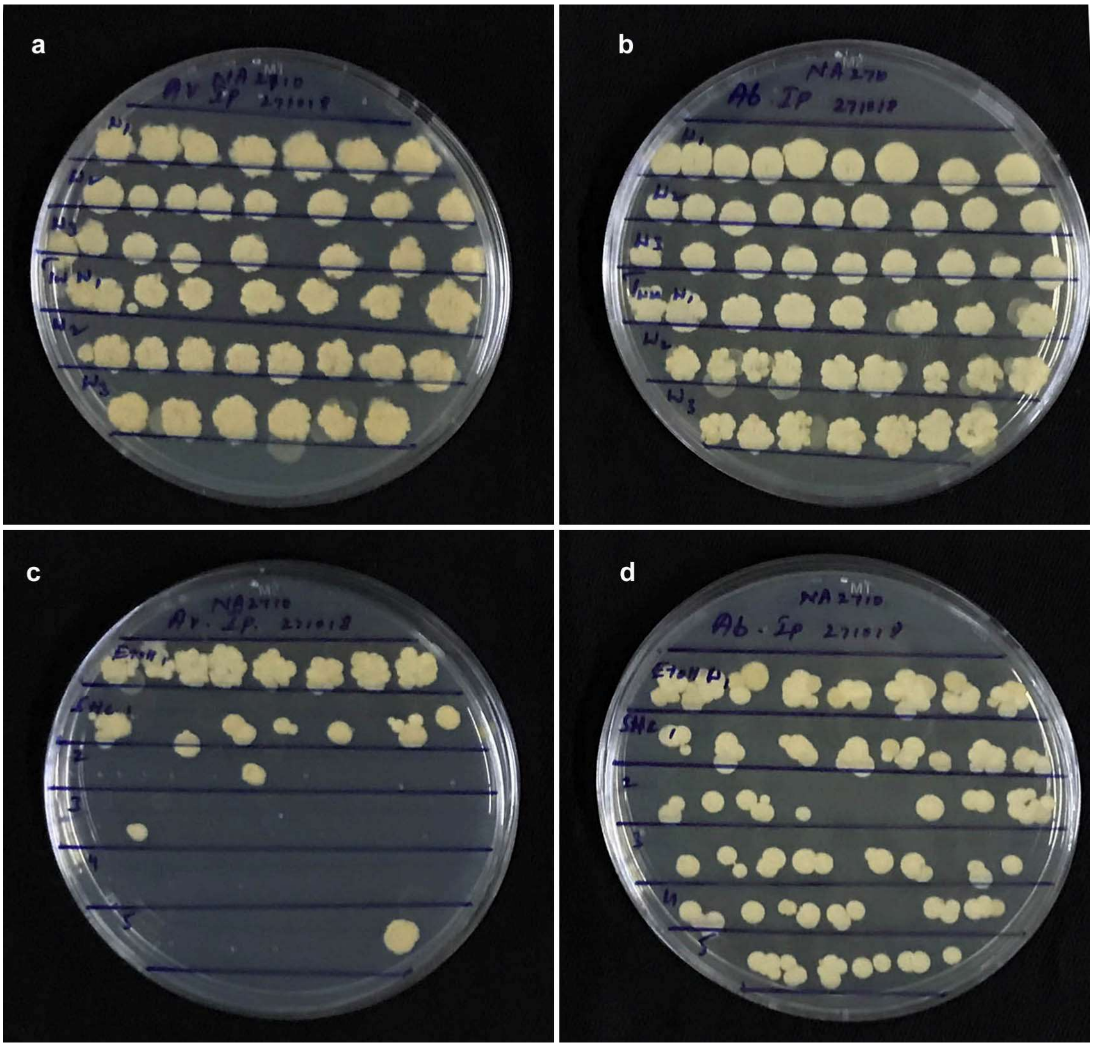

Figure 5. A different seed lot of the tomato 'Arka Vikas' and 'Arka Abha' monitored at surface sterilization through seed wash solution spotting on NA (direct and after Tween-20 step) displaying Bacillus spp./ spore formers (a, b), and inoculum carry over after ethanol wash or NaOCl treatments (c, d) due to difference in the initial bacterial load, or a reduction in the efficacy of the NaOCl.
